# NSCLC as a chaperonopathology: diagnostic and prognostic significance of HSPB5

**DOI:** 10.1186/1749-8090-8-S1-O230

**Published:** 2013-09-11

**Authors:** D Petrov, R Cherneva, G Yankov, D Marinova

**Affiliations:** 1 Thoracic Surgery, Saint Sophia University Hospital for Pulmonary Diseases, Medical University, Sofia, Bulgaria; 2 Pneumology, Alexandrovska University Hospital, Medical University, Sofia, Bulgaria

## Background

Chaperones are essential in carcinogenesis and their non-invasive measurement in peripheral blood of cancer patients is useful for diagnostic and prognostic purposes.

The aim of our study was to determine the role of chaperone HspB5 in tumor biology and assess its significance for NSCLC diagnosis and prognosis.

## Methods

ELISA was applied to determine the plasma levels of HspB5 in 45 patients, operated on for NSCLC, 38 high risk COPD patients and 45 age and sex-matched healthy volunteers. The pTNM of NSCLC patients was defined according to the 7th revision of TNM staging system. All operated on patients were followed up either their death or up to 5 years. ROC analysis was used to characterize the diagnostic role of HspB5. The one and five-year survival rates were defined by Kaplan-Maier.

## Results

Fifteen patients were stage I (IA-5; IB-10), 15 - stage II (IIB-15) and 15 - stage IIIA. HspB5 correlated positively to the local invasion - T factor progression ( T1b -0.309 , T2a – 0.438 ,T2b-0.526 T3 – 0.620, p-0.043). It was useful in distinguishing patients with and without lymphogenic spread (N0-0.369; N1-3 – 0.523, p-0.045). Its plasma levels could discern NSCLC patients (OD-0.413) among high risk COPD patients (0.364) and healthy volunteers (0.276, p-0.001). ROC analysis defined specificity of 72% and sensitivity of 62% at a cut-off 0.317.Neither one, nor five-year survival rates were associated with HspB5 plasma levels (log rank p-0.624)

## Conclusion

HspB5 is positively associated with the local invasion and lymphogenic spread. It may find application in the diagnosis of NSCLC patients, but has no prognostic significance.

